# Sulphanilamide in Treatment

**Published:** 1937

**Authors:** E. Watson-Williams

**Affiliations:** Surgeon-in-charge, Ear, Nose and Throat Department, Bristol Royal Infirmary


### SULPHANILAMIDE IN TREATMENT.

BY

E. Watson-Williams, M.C., Ch.M., F.R.C.S.,

*Surgeon-in-charge, Ear, Nose and Throat Department, Bristol Royal Infirmary.*

---

The introduction of the sulphanilamide group of drugs marks a very definite advance in the treatment of streptococcal and some other infections.

Sulphanilamide is p-aminobenzene-sulphonamide: available for purchase as Prontosil album, Sulphonamide-P, Streptocide. In addition to being efficacious in infection with various types of streptococci, bacillus coli and the typhosus group it is said to be useful against meningococcal infection. Good results have also been claimed in staphylococcal and gonococcal diseases, although there is both clinical and experimental evidence that it has no influence here.

Benzyl-sulphanilamide is p-benzyl-aminobenzene-sulphonamide, Proseptasine. It is claimed to be considerably less toxic than sulphanilamide, and equally effective, at least as regards streptococci. Both drugs are supposed to act directly on the cocci, rendering their capsules more permeable, or at least less protective. The dose of either for an adult is two or three half-gram tablets by mouth thrice daily, though this is often considerably exceeded. Children tolerate proportionate doses well. It is advised that administration should be continued for at least three days after the temperature has become normal: if this is not done there may be a return of symptoms. As a rule it is not given for more than ten days in all. Sulphates (*e.g.* magnesium sulphate) should not be given while it is being administered, for fear of inducing sulphæmoglobinæmia.

Soluble preparations, *e.g.* Prontosil soluble, Soluseptasine, are available for subcutaneous or intramuscular injection in doses of 5 to 20 c.cm. of a half-per-cent. solution. These are complex compounds which liberate sulphanilamide or benzyl-sulphanilamide as the case may be. Intravenous injection is also recommended, but not by me, for I have found that intravenous injection of soluseptasine can produce serious collapse. Intrathecal injection is not advisable. The effect of injection is no greater than that of oral administration, though the former might have value when a rapid effect is wanted : the makers advise oral administration as well. Further combinations are on trial, which it is hoped will be effective against staphylococci and pneumococci.[1]

In my own experience, chiefly with benzyl-sulphanilamide, the most frequent side-effect is a constant mild nausea throughout administration; frequency of micturition may be encountered at the beginning. If drugs of this group are given in too large doses or for too long it is generally recognized that methæmoglobinæmia or sulphæmoglobinæmia may ensue : I have not encountered these complications, which are mentioned in Professor Drew-Smythe's contribution on page 217. Some surgeons, however, maintain that the drug should be pushed until cyanosis from this cause appears, and that such a complication should not be considered a reason for stopping administration : it is difficult to follow the first argument. Mental depression, also, is an effect that I have not noticed. Several cases have been reported of agranulocytosis following the use of sulphanilamide.

My own experience with Prontosil dates from the early months of 1936, during which year I used the drug in seventeen cases. Possibly I was too cautious : at all events I did not observe any very striking results. From the beginning of this year I have used benzyl-sulphanilamide in ninety odd cases. It is fair to say that until recently it was used only in the more serious infections, and the series is far too small for any statistical comparison with cases treated without this remedy. With a great variety of surgical conditions only a very large series of cases is capable of showing the actual value of such an addition, the result in so many being dominated by surgical considerations. For example, I have only used it in three cases of streptococcal meningitis, one of which survived (two are reported below) : this is a smaller proportion of successes than the average of the last ten years. Again, in eleven cases of streptococcal tonsillitis the results have been good, though not strikingly better than those obtained with full doses of sodium salicylate ; but tonsillitis is rarely fatal or even dangerous. In "septic sore throat" or pharyngitis the results are definitely good, and in acute infections of the nasal sinuses and middle ear I have been sufficiently impressed to adopt this as a routine remedy. From the whole series of clinical observations is derived a clear and unequivocal impression that proseptasine is well tolerated and exercises a very beneficial effect in streptococcal infections.

The prophylactic value of this drug does not seem yet to have received attention. A very large proportion of streptococcal infections are traceable to the noses or throats of " carriers " ; and it has become recognized that adequate separation of beds is the surest method of avoiding those epidemics of tonsillitis that occur in wards (at least in nose and throat departments) as soon as the latter are allowed to become overcrowded. I have, happily, not had the opportunity of testing proseptasine in this emergency. But another method of estimating prophylactic value is available, though only convincing where patients are nursed in single rooms. A common complication of nasal sinusitis is acute tonsillitis following operation: it occurs in some 10 per cent. of cases in my own experience. A short series of cases has led me to believe that proseptasine exerts a real influence in protecting the patient against his own streptococcal complications.

Mr. F. W. Willway has been good enough to look up the cases treated with sulphanilamide in the Bristol Royal Infirmary during the past year. Unfortunately, although several good results were obtained, the notes of the eight surgical cases are not sufficiently detailed to reproduce. By the kindness of the physicians in charge, I am able to give a short account of one case of septic pharyngitis and another of streptococcal pyæmia in which these drugs were employed. Notes of a few cases illustrative of my own experience are subjoined.

Dr. B. A. Peters, in a recent publication,[2] summarizes his experiences with proseptasine. In scarlatina the mean duration of pyrexia was slightly greater in those having the drug than in the control series. The total number of complications was about the same in both, but only one-third of the " proseptasine patients " had complications in contrast to over a half of the others ; and the only two deaths occurred in the control series. The results in erysipelas were very striking. "Since this series we have treated a further twenty cases of erysipelas with successful results, that is no death so far in about seventy cases. We have also treated, in addition to those recorded, five cases of puerperal sepsis, and in every case with a rapid and satisfactory recovery. My general impression is that in erysipelas, puerperal sepsis and other hæmolytic streptococcal infections the results are extremely striking; in scarlet fever the results are by no means so dramatic. We are still carrying out further investigations in cases of scarlet fever, but we have not yet enough cases to express any further opinion. I have treated now over three hundred cases with this drug, proseptasine, and have not yet seen any evidence of sulphæmoglobinæmia. We keep our patients on a lacto-vegetarian diet, no eggs, and no sulphur-containing salines, which may account for our favourable results in this respect."*

Sulphanilamide will not work miracles, nor will it replace surgical measures in cases where these are indicated. It is specially necessary to keep this in mind when there are localized collections of pus to be dealt with, for the effect of the drug on these is comparatively poor, though its influence on the general condition may induce a fall in temperature and a general clinical improvement that might otherwise deceive. It is particularly in generalized streptococcal infections, such as erysipelas or septicæmia,[3] that its value is apparent.

Nor do we yet know sufficient about it to adopt it as a routine measure in every case, say, of streptococcal tonsillitis. But all who use it carefully

* Personal communication.

for every severe case of streptococcal infection agree that it constitutes a new and very effective addition to our armamentarium.

**Case 1.**—(Dr. A. T. Todd.) *Streptococcal pyœmia.*

G. H., aged 34. *History :* In bed for one week before admission with a discharging ear and a painful right arm. On admission, high fever; temperature 104·4°F.; painful swelling of right arm; whitlow right thumb; pain in back and chest; diminished movement of both lungs; dullness at both bases, with bronchial breathing which is patchy in distribution. Pathological investigation as follows: swab from ear: streptococci + + +. Blood culture: streptococci grown. Swab from abscess of arm: streptococci in abundance. Urine: sterile. Treatment: tablets Prontosil 2 t.d.s. by mouth. The temperature fell steadily to become normal in eight days: recovery uneventful.

**Case 2.**—(Dr. R. C. Clarke.) *Streptococcal pharyngitis of six days' duration.*

G. N., aged 20. Temperature on admission, 101·6°F. Pulse 108. Respirations 22. Some nasal obstruction but no discharge. Nasal swabs and throat swabs both revealed streptococci in abundance. Œdema under the eyes; crepitations at both lung bases; urine normal. She was treated with Prontosil soluble as follows: an intramuscular injection of 5 c.cm. on the day of admission, repeated on the six succeeding days, when Prontosil was discontinued. She was also given two tablets of Prontosil daily by mouth. She made a steady recovery and was discharged on the twentieth day after admission.

**Case 3.**—*Severe streptococcal pharyngitis ("hospital sore throat").*

Male, aged 28, was admitted very ill with intense congestion of the fauces and some false membrane: temperature 103·6°F. The report on the throat swab was consistently that diphtheria bacilli were absent and streptococci present. He was given 20,000 units of anti-diphtheritic serum when he was first seen and on the second day. When the second pathological report was received 10 c.cm. of soluseptasine was given subcutaneously in the morning and again in the evening. On the third day his throat was materially better and he was able to swallow crushed tablets of proseptasine, three tablets four times a day. By the fifth day his throat was clean and comfortable, his temperature was normal, and he made an extremely rapid recovery.

Case 4.—*Streptococcal mastoiditis with complications.*

A girl of 14 had acute left mastoiditis with nasal sinusitis : she looked pale and puffy (said to be like this always). Temperature 102°F. A mastoid operation was performed with drainage of the right maxillary antrum, but the temperature remained high. She seemed far more ill than one would expect, and on the fourth day she developed acute tonsillitis and the opposite ear began to discharge. She appeared to be putting up a very poor resistance to her streptococcal infection. Proseptasine was administered in doses of two tablets four times a day. After five days she was very much improved, and the temperature was normal. The proseptasine was stopped as she was complaining of constant nausea, but the next day her temperature rose to 100·4° and she felt ill again. The drug was therefore given for a further five days : the temperature become normal on the second of these, and she made a rapid and complete recovery.

Case 5.—*Streptococcal meningitis : death.*

A boy of 10 had mild earache for the first time, without any precedent illness. But he seemed well, eating, playing and sleeping for a week. At the end of the week he again had acute pain in the ear, without the least trace of mastoid swelling or tenderness : temperature 103·0°F. A myringotomy was performed that night : the ear was full of thick pus, which grew only staphylococci. I saw him at noon next day when he had very pronounced rigidity of the neck, positive Kernig's sign, absent knee-jerks, flexor plantar reflex ; photophobia. pupils equal and reacting, fundus veins engorged but no papillœdema ; no headache. A simple mastoid operation was carried out ; the bone was engorged, and there was a little pus in the antrum and in one or two cells ; dura appeared normal. Lumbar puncture produced faintly opalescent fluid under very high pressure : "purulent fluid, 98 per cent. of cells polymorphs, glucose trace, chlorides 0·59 per cent., no bacteria seen" (culture later showed streptococci). Ten c.cm. soluseptasine and 200 c.cm. glucose given intravenously. Next day 50 c.cm. collosol argentum was given intravenously and 10 c.cm. soluseptasine intrathecally

(this caused definite shock): the cerebrospinal fluid was purulent. On the third day he was very ill: silver and soluseptasine were given intravenously (separately); lumbar puncture yielded thick purulent fluid under lower pressure than before, and streptococci were seen in smear preparations. He died that evening. Autopsy: diffuse meningitis, operation site clean.

**Case 6.**—*Streptococcal meningitis: recovery.*

A boy of 15 was seen by me three and a half days after the onset of meningitis, secondary to chronic mastoiditis with suppurative labyrinthitis. The onset had been acute, with vomiting, vertigo and frontal headache; the general and mental states were good and there was no trace of Kernig's sign or of neck rigidity—which was very deceptive. But he had lost his plantar reflexes. Temperature 100·2°F. Lumbar puncture: turbid fluid under high pressure; "purulent fluid, 98 per cent. of cells polymorphs, glucose faintest trace, chlorides 0·66 per cent." Both the duration since onset and the low chloride content were unfavourable features. He was treated by a radical mastoid operation, with labyrinthectomy and drainage of the cisterna pontis via the internal auditory meatus (since with a dead vestibule the probability was that this was the route by which the meninges were infected). He had 15 c.cm. soluseptasine intramuscularly and 50 c.cm. "collosol argentum" intravenously at the operation and took sixteen proseptasine tablets daily. Cerebrospinal fluid drained freely from the wound for four days: the silver injection was repeated on the third, fourth and fifth days. The temperature became normal on the eleventh day, the proseptasine having been stopped on the tenth on account of nausea, and he is making a satisfactory convalescence.

### REFERENCES.

[1] Whitby, L. E. H., *Lancet*, 1937, i. 1,517.

[2] Peters, B. A., *Lancet*, 1937, i. 1,273.

[3] Gibberd, G. F., *Brit. Med. Jour.*, 1937, ii. 695.

